# Improvement of ENU Mutagenesis Efficiency Using Serial Injection and Mismatch Repair Deficiency Mice

**DOI:** 10.1371/journal.pone.0159377

**Published:** 2016-07-21

**Authors:** Jabier Gallego-Llamas, Andrew E. Timms, Rose Pitstick, Janet Peters, George A. Carlson, David R. Beier

**Affiliations:** 1 Center for Developmental Biology and Regenerative Medicine, Seattle Children’s Research Institute, Seattle, WA, United States of America; 2 Department of Pediatrics, University of Washington School of Medicine, Seattle, WA, United States of America; 3 McLaughlin Research Institute, Great Falls, MT, United States of America; Institut de Recherches Cliniques de Montréal (IRCM), CANADA

## Abstract

ENU mutagenesis is a powerful method for generating novel lines of mice that are informative with respect to both fundamental biological processes and human disease. Rapid developments in genomic technology have made the task of identifying causal mutations by positional cloning remarkably efficient. One limitation of this approach remains the mutation frequency achievable using standard treatment protocols, which currently generate approximately 1–2 sequence changes per megabase when optimized. In this study we used two strategies to attempt to increase the number of mutations induced by ENU treatment. One approach employed mice carrying a mutation in the DNA repair enzyme *Msh6*. The second strategy involved injection of ENU to successive generations of mice. To evaluate the number of ENU-induced mutations, single mice or pooled samples were analyzed using whole exome sequencing. The results showed that there is considerable variability in the induced mutation frequency using these approaches, but an overall increase in ENU-induced variants from one generation to another was observed. The analysis of the mice deficient for *Msh6* also showed an increase in the ENU-induced variants compared to the wild-type ENU-treated mice. However, in both cases the increase in ENU-induced mutation frequency was modest.

## Introduction

Forward genetic screens using *N-ethyl-N-nitrosurea* (ENU) as a chemical mutagen have revealed a wide spectrum of biological and disease processes [1]. ENU causes DNA damage by transferring a methyl or ethyl group to the oxygen and nitrogen atoms of nucleotide bases (reviewed in [2] and [3]). The resulting base adducts tend to mispair during semi-conservative replication. If this is not corrected, the following round of replication will convert the mismatch to a point mutation. This method creates random mutations throughout the genome, which are potentially more representative of the mutations responsible for human disease than null or conditional mutations generated by genome targeting.

While mutagenesis screens have been successful in creating a wide range of phenotypes for disease modeling, the efficiency of this approach is still constrained by the number of mutations that can be induced in a single organism. The frequency of ENU-induced mutations is affected by treatment regimens and dosage, but generally ranges from 1–2 mutations/Mb of genomic DNA [4–8]. In a strain such as C57BL/6J, each mutated gamete would carry approximately 3000–6000 mutations, of which around 30–60 mutations would be in coding regions. A major limitation is the dose of ENU that can be administered, as a ceiling is reached such that treatment results in sterility or lethality [9, 10]. As a consequence, ENU screens frequently entail treatment of a large number of animals in order to obtain a mutant phenotype of interest.

A number of different strategies have previously been tested in a bid to increase the number of mutations per animal without decreasing fertility. One involves manipulating DNA mismatch repair (MMR), which is part of the repair mechanism that prevents alkylation damage in cells and protects them from naturally occurring mutations [3, 11, 12]. This machinery corrects small replication errors such as insertion/deletion loops (IDLs) and base-base mismatches. There are five MMR genes in mammals that produce three different heterodimers. The mismatch-bound MutS heterodimer recognizes the replication errors and exists in two varieties: MutSα and MutSβ. MutSα consists of the MMR proteins MSH2 and MSH6, and recognizes single base pair mismatches and small IDLs. MutSβ is a heterodimer of MSH2 and MSH3 and mediates recognition of larger IDLs. Subsequent recognition of the mismatch by MutSα or MutSβ will induce the recruitment of multiple molecules of MutLα. MutLα is the predominant form of the MutL family functioning in MMR, consisting of MLH1 and PMS2 [13].The recruitment of MutLα will activate its endonuclease activity [14] together with the exonuclease EXO1, causing DNA excision to initiate at an upstream nick. The gap created by this excision is repaired by the combined action of proliferating cell nuclear antigen (PCNA), replication factor C (RFC) and DNA polymerase III that functions as its processivity factor. The remaining nick is then sealed by DNA ligase [15].

This mechanism not only recognizes and repairs natural occurring mutations, but has also been shown to be involved in mutations induced by ENU. Claij et al. [16] found that mouse embryonic stem cells lacking one of the components of the MMR, *Msh2*, had a strongly increased mutation frequency when treated with ENU as compared to wild-type. A number of studies have attempted to use the MMR mechanism to increase the number of mutations induced by ENU treatment *in vivo*. Specifically studies were done using *Msh6* mutant zebrafish [17] or rats [18] with different outcomes ranging from no significant increase in zebrafish to a ~2.5 fold increase in ENU-induced variant number in rats. In this study we describe a similar strategy using mice.

An alternative approach to increase ENU-induced mutations would be to use treatment of serial generations, in which ENU injections are administered to the progeny of treated animals. In this report we describe the consequences of both the use of serial injection and the use of an MMR-deficient mouse line. Both methods resulted in increased numbers of mutations detected by exome sequence analysis. However, the maximal increase obtained by either method compared to a single injection was less than 1.8-fold, suggesting these strategies have limited benefit.

## Experimental Methods

### Animals and mutant mouse generation

**Fig 1:**
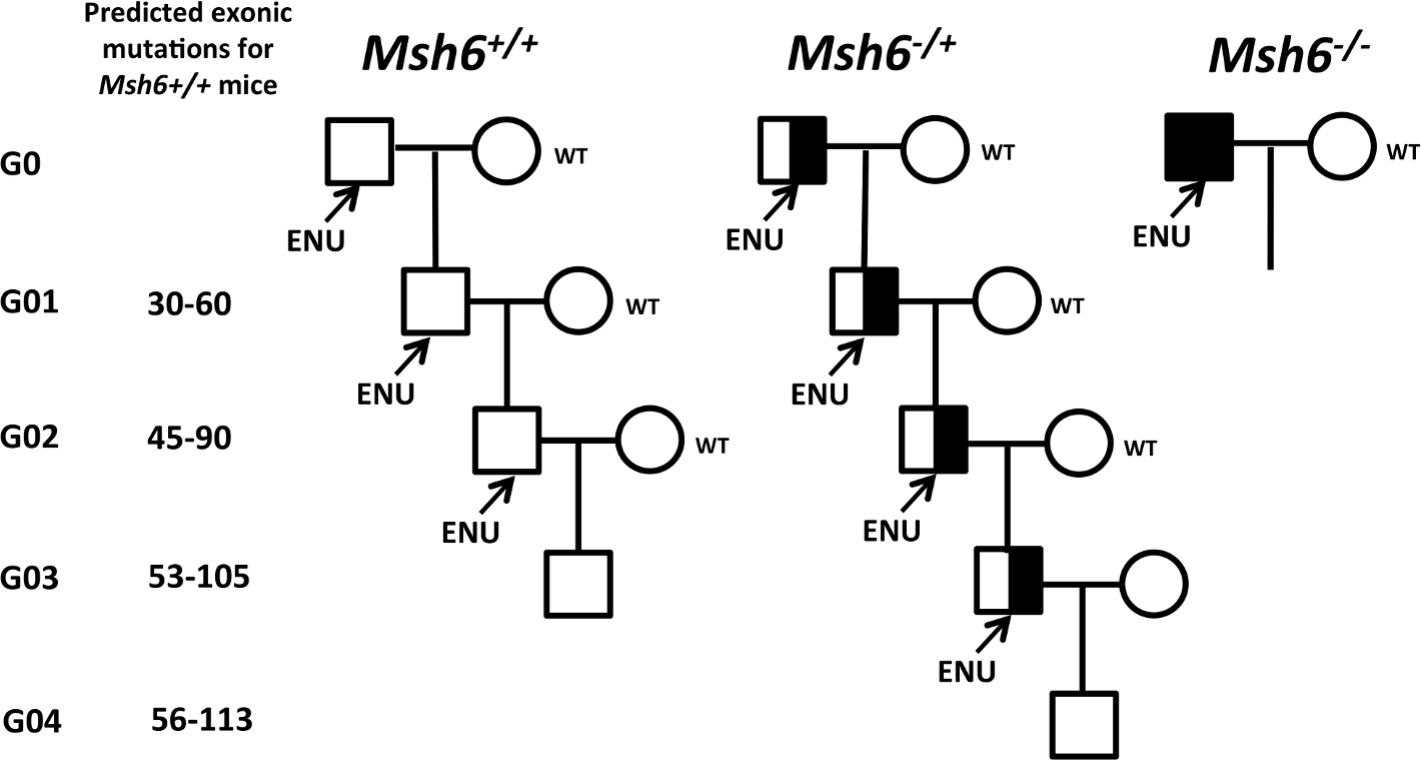
Breeding scheme for analysis of serial ENU-treatment in wild-type and MMR defective mice. G01 males carry a random set of *de novo* point mutations induced by ENU treatment of wild-type or MMR-mutant G0 mice. G02, G03 and G04 carry mutations that were induced in their respective parents, as well as those inherited from previous generations. Each generation treated with ENU will on average have 3000–6000 new ENU-induced variants genome wide. After ENU treatment each male is crossed with a wild-type female; their progeny will inherit newly induced mutations and 50% of those of the parent. The mutations are sampled by exome analysis; the expected number of ascertained mutations (E) is shown. The ENU treatment was performed on three successive generations for the *Msh6*^*+/+*^ mice and four generation for the *Msh6*^*+/-*^ mice. *Msh6*^*-/-*^ mice did not tolerate ENU treatment.

All animals were bred and maintained under specific pathogen-free conditions at McLaughlin Research Institute’s AALAC International accredited Animal Resource Center. All protocols were reviewed and approved by the Institutional Animal Care and Use Committee. The *Msh6* mutant line *Msh6*^*tm1Rak*^ [19] was kindly provided by Dr. Winfred Edelmann. Wild-type (*Msh6*^*+/+*^), *Msh6*^+/-^, and *Msh6*^-/-^ mice were treated with ENU as previously described (reviewed in [20]). Briefly, adult male animals (Generation 0 (G0)) were treated with three weekly intraperitoneal injections of 90mg of ENU per kilogram of body weight. Treated males were individually housed with ICR females to test sterility and the time to recovery of fertility. After fertility was recovered, the animals were mated with C57BL/6J (B6) females to generate G01 offspring (Fig 1, Table 1). The G01, G02, and, from the *Msh6+/-* cohort, G03 progeny males were treated with the same ENU regimen. Females from the different generations (G01, G02, G03, and G04) and mutation cohorts were sequenced. Given the various breeding patterns (see S1 Fig for an example), the relatedness of mice in the different cohorts was not identical.

**Table 1:** Fertility after ENU treatment.

		Genotype x dose^1^			
Generation		Msh6^+/+^ x 90	Msh6^-/+^ x 90	Msh6^-/-^ x 90	Msh6^-/-^ x 75
G01	# males injected	17	31	8	4
	# recovering fertility^2^	8	14	0	0
	total # of progeny	142	198	0	0
G02	# males injected	18	30		
	# recovering fertility	11	22		
	total # of progeny	101	140		
G03	# males injected	21	22		
	# recovering fertility	16	17		
	total # of progeny	118	248		
G04	# males injected		15		
	# recovering fertility		9		
	total # of progeny		117		

^1^Dose is indicated as mg of ENU injected per Kg bodyweight x 3 injections.

^2^Fertility is measured by presence at least of one litter within 10 weeks after the last injection.

Mice treated with ENU are at risk for developing tumors and were observed every work day for signs of distress, including wasting, hairloss, inactivity, mass formation, etc. Mice identified as possibly having a tumor were euthanized using carbon dioxide narcosis, followed by cervical dislocation and bilateral thoracotomy, consistent with the recommendations of the American Veterinary Medical Association (AVMA) Guidelines on Euthanasia. No mice were found dead. As mice were euthanized immediately upon showing signs of illness, no other methods were taken to ameliorate suffering.

### DNA extraction

We isolated DNA from liver by phenol/chloroform extraction or using a Qiagen DNeasy Blood and Tissue Kit (Qiagen, Santa Clarita, CA USA). Individuals or pools of 6 to 10 samples of extracted DNA for each generation treated with ENU were then sent for library preparation and whole exome sequencing at Centrillion Genomics Technology (CA, USA).

### Sequencing data analysis

We mapped paired end sequencing data to the reference genome mm10 using BWA-MEM (Burrows-Wheeler alignment tool)[21] employing default parameters. We used the Genome Analysis Toolkit (GATK)[22] to realign reads around known indels, and recalibrate quality scores to reduce sequencing artifacts. Picard (Broad Institute: http://broadinstitute.github.io/picard/) was used to identify duplicate reads. SNPs were identified using SAMtools (mpileup, bcftools)[23]. SNPs were annotated with ANNOVAR [24] employing refGene as the gene model. To control for differences in the amount of sequence per sample we identified all regions covered by either 20 reads (individual mice) or 100 reads (pooled mice) using generated Python scripts and the Python version of BEDTools [25] (Pybedtools) [26]. We applied additional filtering to identify ENU induced SNPs with the highest confidence. SNPs in publically available databases (Sanger Mouse exomes, dbSNP) or in-house controls, located within a repeat region, or with low likelihood of being real (<Q30) were removed. Sequence data have been deposited in the SRA repository with the accession number SRP074832. Statistical analyses were done using two-tailed T-tests assuming unequal variances.

## Results

**Fig 2:**
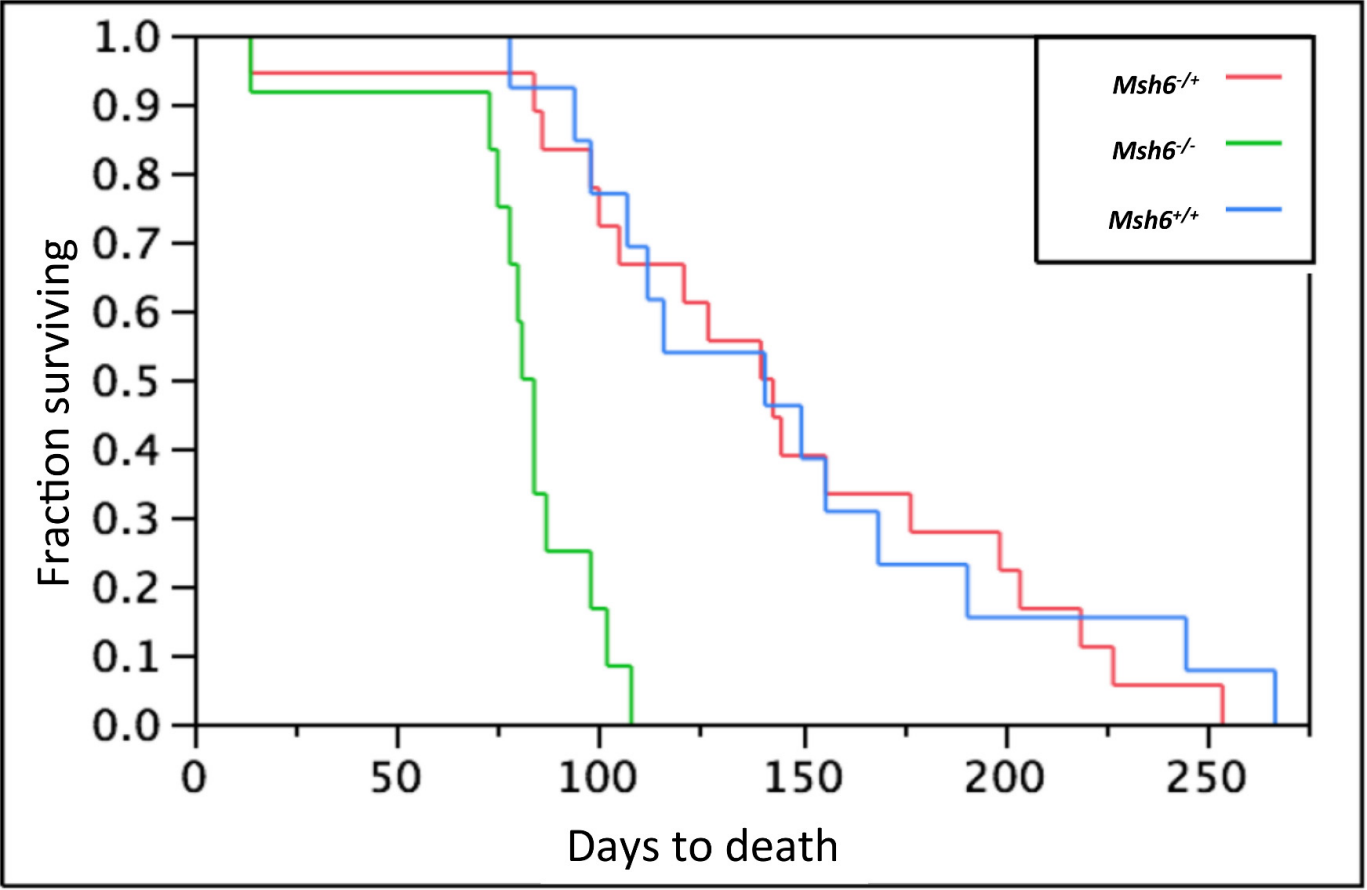
Effect of ENU treatment on survival. *Msh6*^*+/-*^ males show survival comparable to wild-type males after treatment with the standard concentration of ENU (90mg/kg x 3). Survival of ENU-treated *Msh6*^*-/-*^males is much reduced. Death is expressed in days.

ENU treatment was done on 3 serial generations of B6 wild-type and 4 serial generations of B6 *Msh6*^*+/-*^ mice (Fig 1). We initially also included *Msh6*^*-/-*^ mice, but they did not tolerate ENU treatment (Fig 2) even at a reduced dose (Table 1), as they developed thymic tumors with enlarged lymph nodes and spleens (data not shown). Wild-type and *Msh6*^*+/-*^ mice tolerated the ENU administration and more than half of injected mice recovered fertility to allow the production of offspring (Table 1). Serial ENU administration to the progeny of the ENU-treated animals had no cumulative effect on fecundity as after each round of injection there was no decrease in the number of animals that recovered fertility and produced progeny (Table 1).

The efficiency of the ENU treatment for inducing mutations has generally been reported to range from 1–2 mutations/Mb. Half of the autosomal mutations present in G01 mice will be transmitted to the next generation. Therefore, in the second treated generation there should be a 1.5 fold increase in mutation frequency relative to the first treated generation and in the third generation there should be a 1.75 fold increase. We analyzed the number of variants present in each of 3 individual mice from 3 generations of wild-type or *Msh6*^*+/-*^ mice using whole exome sequencing. This strategy was chosen as it enabled us to sample the genome with high confidence and reproducibility, allowing reliable comparison of the cohorts. On average there was an increase in the number of variants present in the next generation for both backgrounds of mice, albeit less than expected (Table 2). Notably, individual mice from the same cohort had very different variant numbers (Table 2 and S1 Fig). Given this variability and the small number of samples per cohort, the differences in mean variant number do not reach statistical significance.

**Table 2:** Variant analysis for individual mice.

Genotype and Generation	variant for each individual			average	ratio^1^	ratio H/W^2^
wild-type G01	86	60	72	73	1.0	
wild-type G02	103	73	54	77	1.1	
wild-type G03	110	118	53	94	1.3	
*Msh6*^*+/-*^ G01	70	77	49	65	1.0	0.9
*Msh6*^*+/-*^G02	135	106	57	99	1.5	1.3
*Msh6*^*+/-*^ G03	108	56	124	96	1.5	1.0

^1^Ratio: the increase of variant present compared to the first ENU injection (G01)

^2^Ratio H/W: the increase of variant present in *Msh6*^*+/-*^ compared to the same wild-type generation.

To obtain a more robust representation of the ENU-induced mutation frequency, we analyzed a pool of 10 mice for each cohort (except for the *Msh6+/-* G04 pool, which was 6 mice). While this has the virtue of including more samples, coverage of each sample was on average 10X (compared to 20X for the individual analysis), so the number of mutations per exome ascertained was smaller. However, this sample depth still enabled comparison among cohorts. The variant number in each pool increased after each generation as previously observed, except for the fourth generation of ENU-treated *Msh6*^*+/-*^ mice (Table 3). However, also as previously observed, the increase in mutation frequency was lower than predicted.

**Table 3:** Variant analysis for pooled mice.

Genotype and Generation	variants	ratio^1^	ratio H/W^2^
wild-type G01	186	1.00	
wild-type G02	259	1.39	
wild-type G03	295	1.59	
*Msh6*^*+/-*^ G01	256	1.00	1.38
*Msh6*^*+/-*^G02	313	1.22	1.21
*Msh6*^*+/-*^ G03	325	1.27	1.10
*Msh6*^*+/-*^ G04	311	1.21	

^1^Ratio: the increase of variant present compared to the first ENU injection (G01)

^2^Ratio H/W: the increase of variant present in *Msh6*^*+/-*^ compared to the same wild-type generation.

An aim of this experiment was to test whether treating mice at least partially deficient in *Msh6* would increase the mutagenesis frequency. In the individually analyzed mice, only one of the 3 generations showed an increase of mutation frequency in the *Msh6*^*+/-*^ group, and this was small (Table 2). Again, the variability within a cohort was problematic. The analysis of pools of mice revealed a marked increase in the variant number for the *Msh6*^*+/-*^ mice compared to the wild-type mice (Table 3). The mutation frequency was further increased for the second (G02) and third generations (G03); however, the ratio of mutations compared to the wild-type cohort decreased for each successive generation.

**Fig 3:**
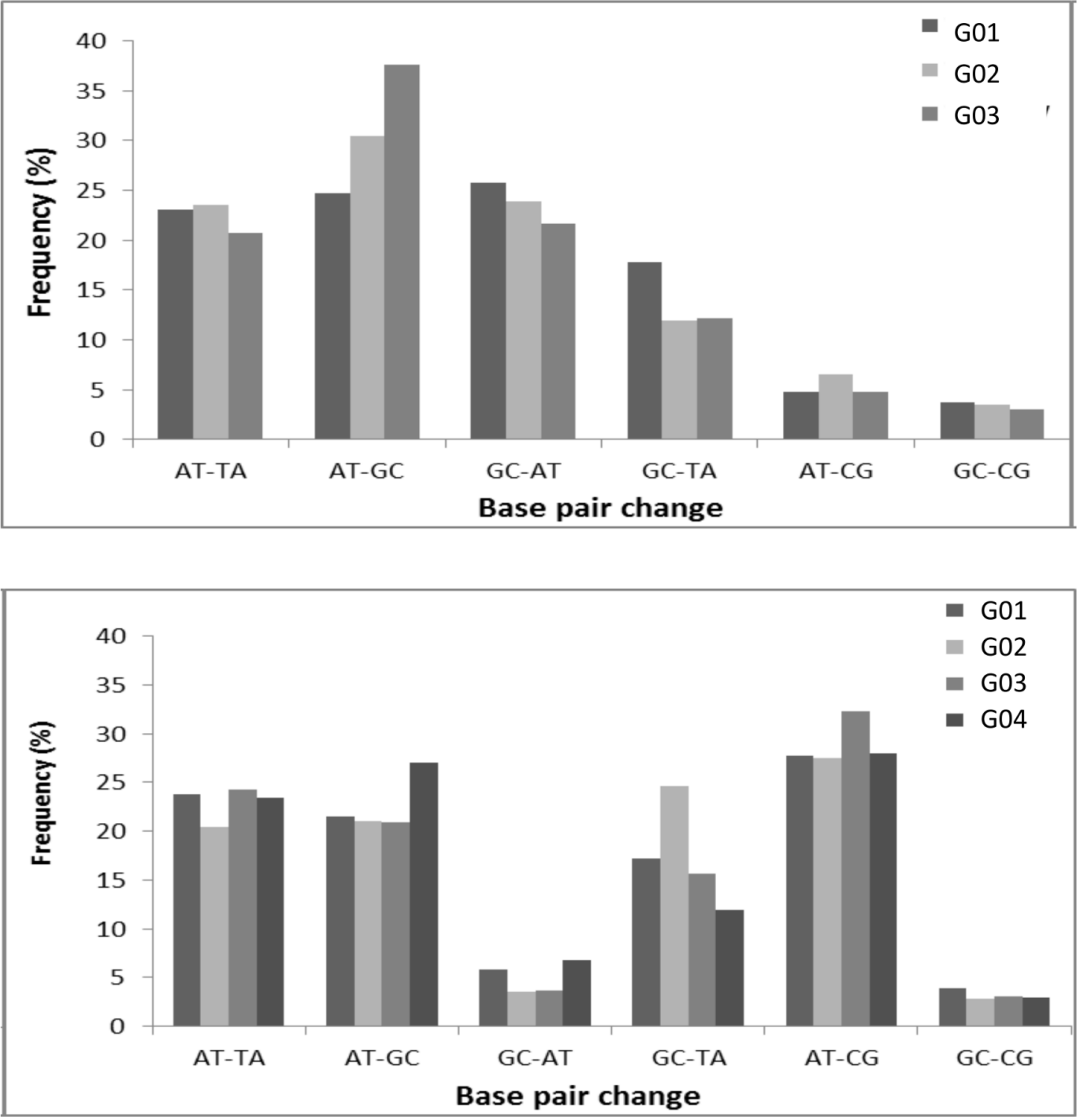
ENU-induced mutation spectrum. (a) Frequencies of mutations in successive generation of *Msh6*^*+/+*^ mice in the pooled samples. (b) Frequencies of mutations in the successive generation of *Msh6*^*+/-*^ mice in the pooled samples.

Examination of the type of mutations produced by the ENU treatment revealed a different spectrum in the *Msh6*^*+/-*^mice compared to wild-type. The most frequent base-pair alterations induced by ENU affected A-T base pairs, with A-T to T-A and A-T to G-C substitutions accounting for almost fifty percent of the mutations for both the wild-type and *Msh6+/-* ENU treated mice (Fig 3), which is consistent with previous results [27]. However, G-C to A-T transitions were much reduced in the *Msh6*^*+/-*^mice compared to wild-type mice, while A-T to C-G transitions were elevated (Fig 3). Overall, wild-type mice have a similar fraction of A-T and G-C modifications, in contrast to the *Msh6*^*+/-*^ mice that had an average of 75% A-T modifications compared to 25% G-C modifications (p = 0.015, Table 4).

**Table 4:** Distribution of ENU-induced mutations in A-T vs. G-C base-pairs.

	%A-T	%G-C	Avg %A-T	Avg %G-C
wild-type G01	53	47		
wild-type G02	61	39	59	41
wild-type G03	63	37		
*Msh6*^*+/-*^ G01	73	27		
*Msh6*^*+/-*^G02	69	31		
*Msh6*^*+/-*^ G03	78	22	75	25
*Msh6*^*+/-*^ G04	78	22		

## Discussion

In this study we demonstrate that serial administration of ENU to successive generations of mice increases the ENU-induced mutation frequency in both wild-type and *Msh6*^*+/-*^mice. Our initial analysis of 3 individual mice in each of 3 generations supported this observation; however, we found considerable variation among individuals. We therefore analyzed pooled DNA obtained from up to 10 different mice from each generation, which confirmed this observation.

The increase we observed was less than expected based on Mendelian inheritance, which could be explained in a number of ways. For example, we used stringent parameters for whole exome sequencing analysis in order to increase the likelihood that we identified *de novo* ENU-induced variants; however, these may have excluded some true variants These parameters could be modified but the filtering of false positives would be less efficient. Another possibility is that we are not accounting for loss due to lethality; however, given that even null mutations of important developmental genes are generally well tolerated in mice when heterozygous, this is unlikely to be an important factor. Of note, this is the case for individual mutant loci; it is possible that an accumulation of heterozygous mutants could affect viability or fertility.

We and others have hypothesized that a deficiency in the repair system that detects or corrects DNA mismatches would result in an increased ENU-induced mutation frequency. This is based on the biochemistry of the mutagen ENU, which can transfer its ethyl group to oxygen or nitrogen radicals present in DNA; this results in lesions that can cause mispairing during replication and eventually give rise to a single base pair substitution[28, 29]. Three other studies addressed this question in ES cells and mice [16], zebrafish [17] and rats [18].

In an *Msh2* null ES cell line, the background mutation rate (assayed by analysis of loss of HPRT activity) was elevated at least two orders of magnitude compared to wild-type cells, and the mutation rate after ENU treatment was further increased 2–3 fold [16]. In an *Msh2*-deficient line that had 10% of wild-type activity, the mutation rate in untreated cells was close to background, and was increased 7–10 fold after ENU treatment. While this would seem promising for a strategy of mutagenesis in an MMR deficient mouse, it is difficult to compare the dose regimen of ENU treatments for *in vitro* and *in vivo* studies, and it is possible the effective dose given in these studies would not be tolerated *in vivo*. Indeed, in our hands *Msh6*^*-/-*^ mice did not tolerate even low doses of ENU.

In *Msh6*^*-/-*^ rats, treatment with ¾ of a dose used in wild-type was modestly successful, elevating the mutation rate to 0.8 mutations/Mb, a 1.5-fold increase compared to the mutation rate in *Msh6*-proficient animals [18]. *Msh6*^*+/-*^ rats were not tested. In contrast, in zebrafish there were no differences found for the mutation frequency for ENU-treated wild-type, *Msh6*^*+/-*^ and *Msh6*^*-/-*^ lines [17]. The authors speculate that these results may indicate that the maximum mutation load for zebrafish has been reached with the currently used, highly optimized ENU mutagenesis protocol; it is notable that the mutation rate obtained of 6–7 mutations /Mb is 3–6 fold greater than that generally obtained in ENU-treated mice. Alternatively, the authors suggest that the MMR system in the zebrafish germ line may be saturated very rapidly, thereby having a limited effect on high-dose ENU mutagenesis.

In our analysis we see a small increase in mutation frequency in treated *Msh6*^*+/-*^ mice compared to wild-type for both individually analyzed and pooled cohorts. In the latter, which likely represents a more representative sample, after 3 serial treatments 325 variants were detected in 6 Mb obtained from 10 mice, for a mutation rate of 5.4 mutations/Mb per mouse. This is 1.1-fold the rate in an equivalent wild-type cohort, and 1.75-fold the frequency found in a wild-type cohort after a single treatment.

Our results also demonstrate that the common mutation both in wild-type and *Msh6*^*+/-*^ background were alterations of A-T base pairs, with *Msh6*^*+/-*^ having a higher ratio of A-T transitions. These results are consistent with what was observed in the *Msh6*-deficient rats and the *Msh2*-deficient mice [16, 18] where they also observed a decrease of A-T to G-C transitions. This phenomenon was associated with the fact that ethylated bases have been shown to direct misincorporation of bases when DNA polymerases succeed in trans-lesion synthesis [30, 31]. Bypass of O^2^-ethylthymineT subsequently induced A-T to T-A transversions, whereas O^4^-ethylthymine causes A-T to G-C transitions [30, 31]. These DNA adducts contribute to the toxicity and mutagenicity of ENU and are speculated to be the target of MMR activity [16]. It has been suggested that MutSα preferentially recognizes the O^2^-ethylthymineT lesion [18], which is consistent with our observation that transversions are elevated in the *Msh6+/-* mutants at the expense of transitions.

There are a number of possible explanations for why the strategies we tested failed to substantially elevate the mutation frequency induced by ENU. One possibility for the limited effect of reducing *Msh6* gene dosage is that 50% of enzyme was sufficient for normal DNA repair. This is clearly not the case, as the spectrum of mutations in the *Msh6*^*+/-*^ cohort is different than in wild-type, indicating that this reduction has biological consequences. It also suggests the possibility that more comprehensive testing in homozygous *Msh6*^*-/-*^ mice might identify an ENU dose that is compatible with survival but is still mutagenic. However, it should also be noted that there are other DNA damage repair pathways that perform partially redundant functions with MMR. For example, the nucleotide excision repair pathway has been shown to mediate ENU-induced mutation repair in flies [32]. One could imagine mutagenizing mice carrying multiple DNA repair defect mutations, although the husbandry task could potentially outweigh the benefit.

The results also suggest the possibility that our protocols do not succeed because we reached a ceiling on the level of mutations that mice can tolerate. While intriguing, this seems unlikely for a number of reasons. Firstly, one would require treating multiple additional generations to insure that the mutation frequency has truly peaked. Secondly, the mutations generated in our strategy are heterozygous (as we outcross at every generation), and these are generally well-tolerated in mice, even when the homozygous phenotype is lethal. Lastly, we see no obvious effect on fertility, fecundity, gross morphology, or general viability, which one might expect if there were additive effects of multiple mutations. Determining what the upper limit of heterozygous mutational load might be in a mammalian system would be interesting, but we do not think this study conclusively addresses this.

The wealth of studies using ENU mutagenesis for a wide variety of phenotypic analyses demonstrates unequivocally that this approach is efficient, that these mutations can be readily mapped and that they can ultimately be identified using well-established approaches for positional cloning [33]. However, given the development of powerful new methods for generating targeted mutations [34,35,36], it is necessary to examine the utility of an approach that requires positional cloning for mutation discovery. **I**n this regard, the expedience of a genotype-driven approach must be balanced against that of a phenotype-driven method, in which one may obtain unbiased insight into the genetic basis of a trait. Many studies employing forward genetic analysis have provided unexpected insights and have identified mutations in unannotated genes whose function were otherwise unknown.

Importantly, the mutations induced by ENU often have functional consequences that are more informative than the null mutations that are commonly generated by genomic targeting. ENU induced mutations often create hypomorphic alleles, due either to “leaky” splice-site mutations or due to missense mutations that do not completely abolish gene function. Missense mutations induced by ENU can also result in dominant “gain-of-function” effects, which can provide insight into protein structure-function and roles in specific developmental pathways that are not revealed by characterization of null mutants.

In summary, the two strategies that we have described in this study do increase the ENU mutagenesis efficiency. However, the increase is small, and can arguably be equaled by simply expanding a screen that employs a standard treatment for a single generation. It is possible that the small increase reflects a biological limit on the number of heterozygous mutations that are tolerated in mice, but this would require additional analysis.

## Supporting Information

S1 Fig Pedigree from one family of mice used for the pooled sample analysis. The G0 through G04 generations are shown. *Msh6*^*+/-*^ heterozygotes are shown as half-filled symbols. Mice used for DNA sample preparation are shown in red. For the total pool mice were obtained from multiple pedigrees, and relatedness of those used was not identical across generations.
(TIFF)

S2 Fig ENU induced point mutation frequencies in individual mice. G01, G02, and G03 samples of W (*Msh+/+*) and H (*Msh+/-*) mice are shown.
(TIFF)
