# Supplementary figures and images for: Improvement of ENU Mutagenesis Efficiency Using Serial Injection and Mismatch Repair Deficiency Mice
Source: PLoS One. 2016 Jul 21;11(7):e0159377. doi: 10.1371/journal.pone.0159377 (PMC4956170; doi:10.1371/journal.pone.0159377)

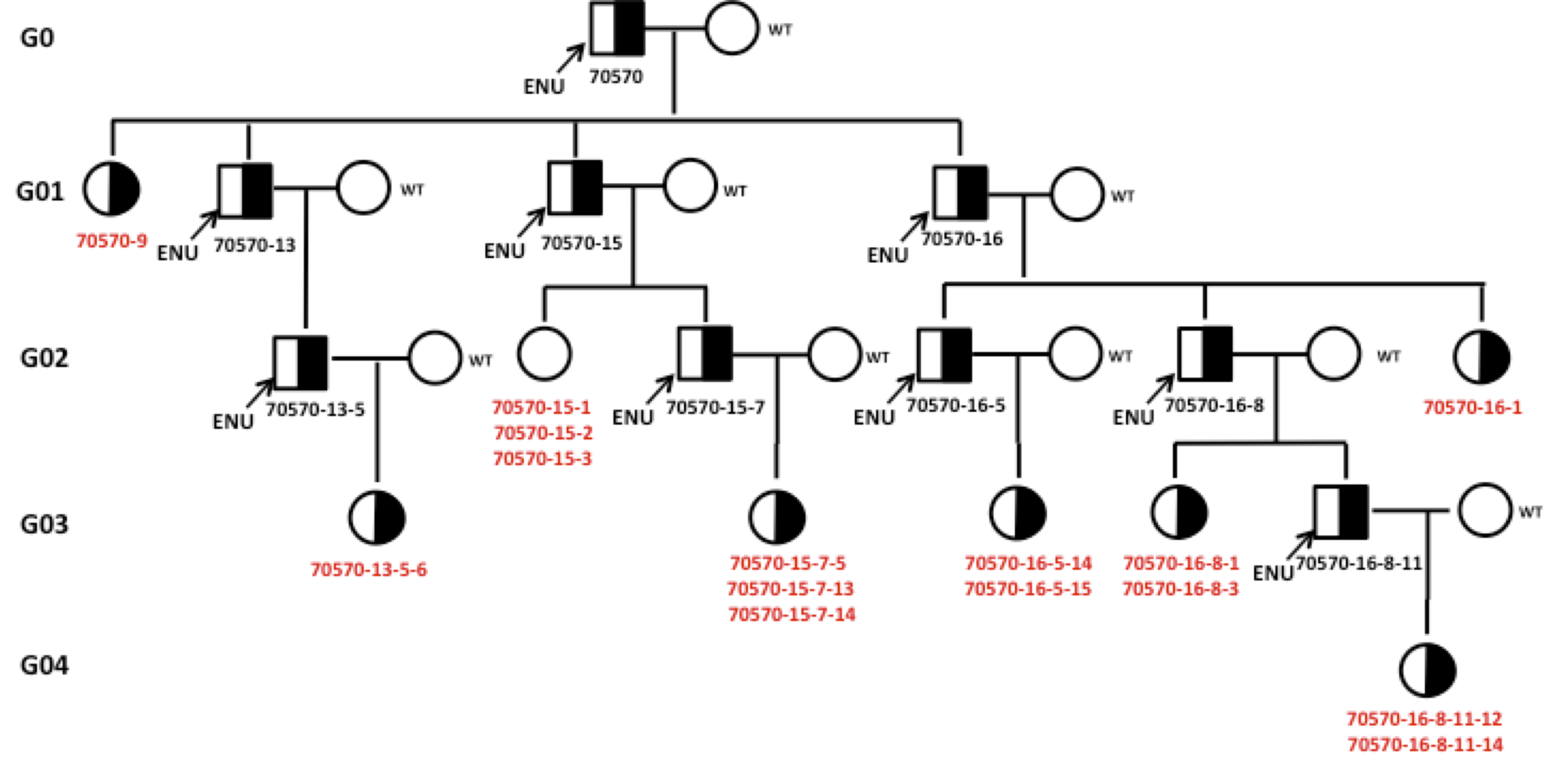

Supplement: S1 Fig — The G0 through G04 generations are shown. Msh6+/- heterozygotes are shown as half-filled symbols. Mice used for DNA sample preparation are shown in red. For the total pool mice were obtained from multiple pedigrees, and relatedness of those used was not identical across generations. (TIFF) [file pone.0159377.s001.tiff]

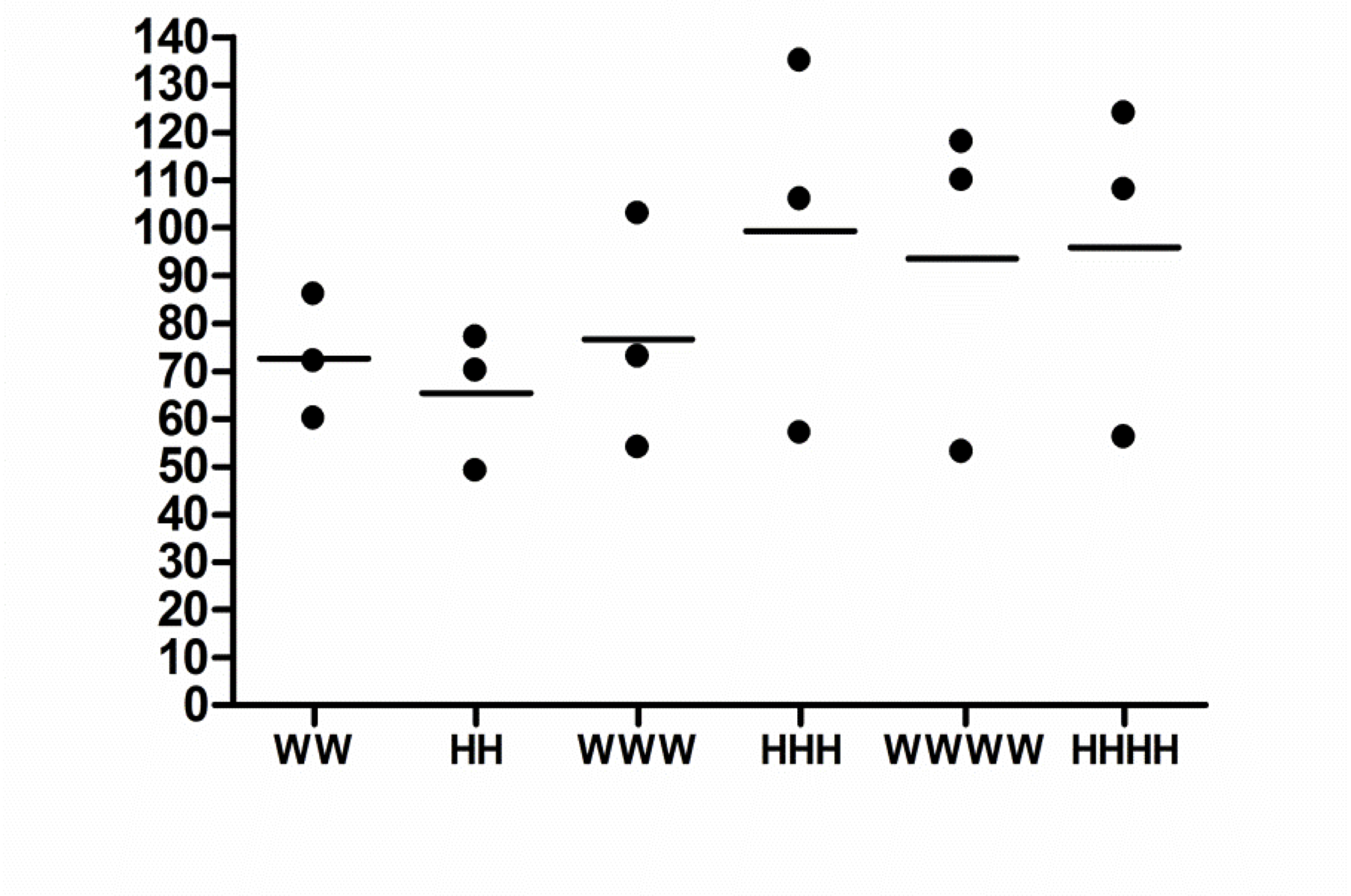

Supplement: S2 Fig — G01, G02, and G03 samples of W (Msh+/+) and H (Msh+/-) mice are shown. (TIFF) [file pone.0159377.s002.tiff]
